# Automated machine learning for image-based detection of dental plaque on permanent teeth

**DOI:** 10.3389/fdmed.2024.1507705

**Published:** 2024-11-28

**Authors:** Teerachate Nantakeeratipat, Natchapon Apisaksirikul, Boonyaon Boonrojsaree, Sirapob Boonkijkullatat, Arida Simaphichet

**Affiliations:** ^1^ Department of Conservative Dentistry and Prosthodontics, Faculty of Dentistry, Srinakharinwirot University, Bangkok, Thailand; ^2^ Faculty of Dentistry, Srinakharinwirot University, Bangkok, Thailand

**Keywords:** automated machine learning, image classification, dental plaque, digital health, Vertex AI

## Abstract

**Introduction:**

To detect dental plaque, manual assessment and plaque-disclosing dyes are commonly used. However, they are time-consuming and prone to human error. This study aims to investigate the feasibility of using Google Cloud's Vertex artificial intelligence (AI) automated machine learning (AutoML) to develop a model for detecting dental plaque levels on permanent teeth using undyed photographic images.

**Methods:**

Photographic images of both undyed and corresponding erythrosine solution-dyed upper anterior permanent teeth from 100 dental students were captured using a smartphone camera. All photos were cropped to individual tooth images. Dyed images were analyzed to classify plaque levels based on the percentage of dyed surface area: mild (<30%), moderate (30%–60%), and heavy (>60%) categories. These true labels were used as the ground truth for undyed images. Two AutoML models, a three-class model (mild, moderate, heavy plaque) and a two-class model (acceptable vs. unacceptable plaque), were developed using undyed images in Vertex AI environment. Both models were evaluated based on precision, recall, and F1-score.

**Results:**

The three-class model achieved an average precision of 0.907, with the highest precision (0.983) in the heavy plaque category. Misclassifications were more common in the mild and moderate categories. The two-class acceptable-unacceptable model demonstrated improved performance with an average precision of 0.964 and an F1-score of 0.931.

**Conclusion:**

This study demonstrated the potential of Vertex AI AutoML for non-invasive detection of dental plaque. While the two-class model showed promise for clinical use, further studies with larger datasets are recommended to enhance model generalization and real-world applicability.

## 1 Introduction

**Figure 1:**
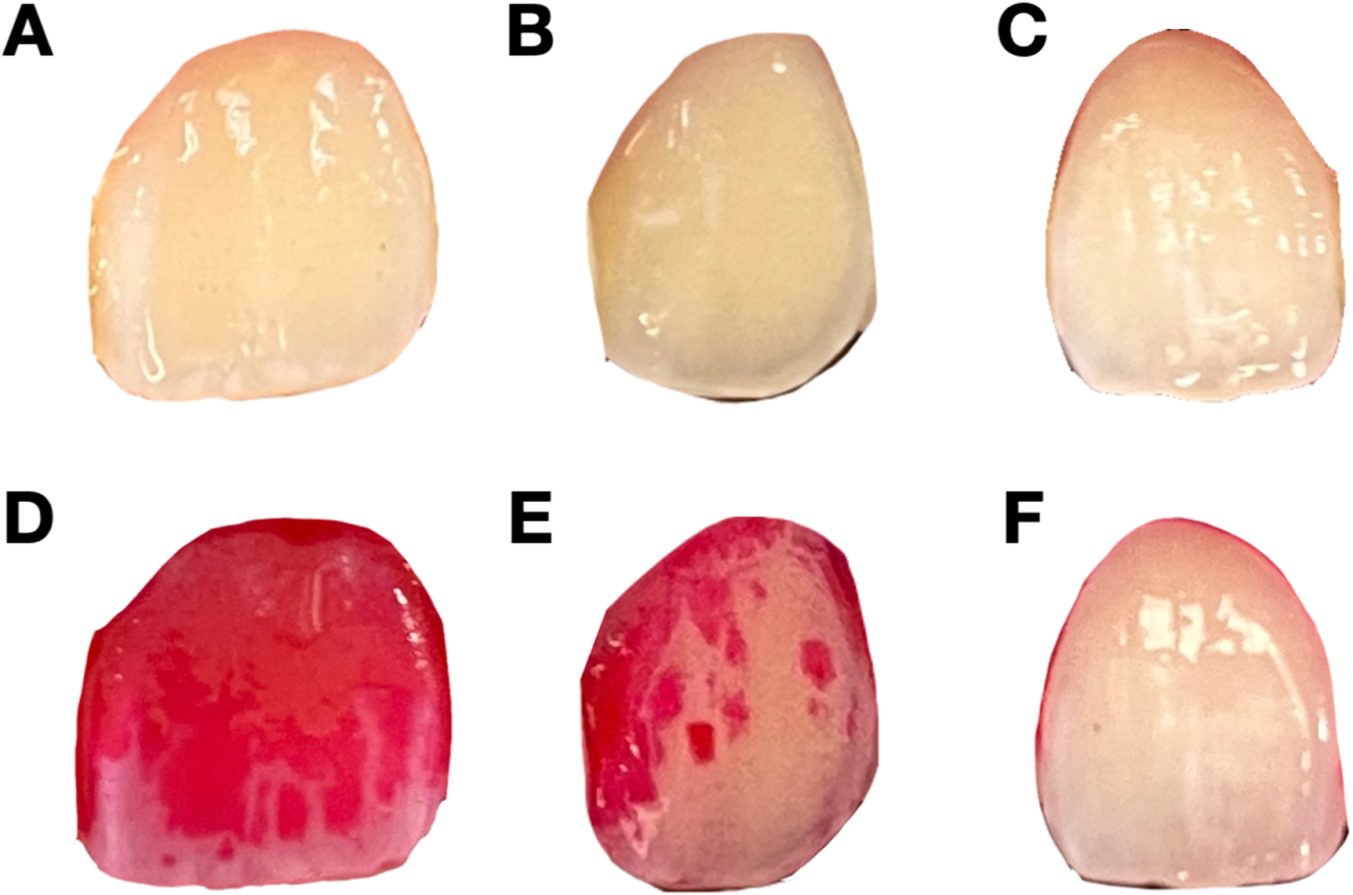
Representative undyed and dyed images of teeth with different levels of dental plaque deposit. **(A–C)** Undyed images of teeth with varying levels of plaque accumulation. These images are used for the model training to predict plaque levels without dye application. **(D–F)** Corresponding dyed images after the application of erythrosine dye, showing the presence and extent of dental plaque. **(D)** represents heavy plaque coverage, **(E)** represents moderate plaque coverage, and **(F)** represents mild plaque coverage.

Dental plaque, a sticky substance composed of bacteria that forms on tooth surfaces, significantly contributes to the development of periodontal diseases. If dental plaque is not adequately removed, it can lead to the destruction of periodontal tissues and ultimately result in tooth loss (1). Therefore, detecting and controlling of dental plaque are crucial steps in preventing periodontal disease (2). Conventionally, dental plaque is detected by probing around the gingival margin with an explorer (3). However, as shown in Figures 1A–C, without dyes, it is difficult to evaluate the level of dental plaque with the naked eye. To enhance visualization of the dental plaque and to motivate patient cooperation, erythrosine solution is commonly used for dyeing areas of dental plaque accumulation because it specifically stains the surface deposits, making dental plaque visibly distinct from the surrounding tooth surface (4, 5). While effective, the use of plaque-disclosing dyes has limitations. They can temporarily stain the oral mucosa and lips, which raises significant esthetic concerns. Their use also requires specific disclosing agents and techniques, making home-use products challenging for patients to apply and interpret accurately. Additionally, manual plaque assessment in clinical settings is time-consuming and subject to human error, especially in busy environment. To address these challenges, there have been many efforts to use digital technologies for dental plaque detection, such as three-dimensional imaging using intra-oral scanner and fluorescence-based methods. However, there are still many limitations in real-world application, including requirement of specific staining solutions and equipment (6). These problems highlight the need for a more convenient and automated method of dental plaque detection.

Given the limitations of current methods, machine learning, a subset of artificial intelligence (AI), offers a promising alternative for automating diagnostic tasks in healthcare. Machine learning models have been successfully applied to various healthcare applications (7). However, developing effective machine learning models requires significant technical expertise, which is challenging for researchers and clinicians who are not knowledgeable in data science (8). Automated machine learning (AutoML) platforms, like Google Cloud's Vertex AI simplify the process of machine learning models and enable users with limited machine learning knowledge to create accurate models (9). AutoML, particularly image-based models, has been increasingly applied in various healthcare settings, demonstrating its ability to assist in the diagnosis of conditions like retinal diseases and lung cancer (10, 11).

In the field of dentistry, the use of machine learning is still in the early stages, but there is growing interest in utilizing these technologies for diagnostic and therapeutic purposes (12). For dental plaque detection, some current studies have reported the use of machine learning models to detect dental plaque areas from various sources of images. Although the reported models demonstrated the good accuracy, they typically required the technical expertise to develop the training algorithm, which may limit the future development of AI-assisted models by clinicians (13–15). These challenges highlight the advantages of AutoML to be used to develop the automated models. However, to the best of our knowledge, the application of AutoML for the detection of dental plaque, particularly using photographic images for level classification remains relatively unexplored. Given the potential of AutoML, there is a need to investigate its capability for detecting dental plaque by classification tasks through automated systems as an alternative to traditional dye-based methods.

In this study, we aimed to investigate the feasibility of using Google Cloud's Vertex AI AutoML to develop an automated model for detecting dental plaque levels on permanent teeth using undyed photographic images. This approach offers a non-invasive, scalable, and accessible solution for both clinicians and patients.

## 2 Materials and methods

### 2.1 Study approval and participant criteria

The study was approved by The Human Research Ethics Committee of Srinakharinwirot University (SWUEC-671045), and informed consent was obtained from all participants. One hundred dental students at Faculty of Dentistry, Srinakharinwirot University, Bangkok, Thailand were enrolled in this study. Participants were required to have the permanent upper anterior teeth (teeth 13–23) without any restoration or fixed appliance.

### 2.2 Data collection

Photographic images of the permanent upper anterior teeth were taken with a 12-megapixel smartphone camera (iPhone 13, Apple Inc, Cupertino, CA). Standardized photographic protocols were implemented to ensure consistency across all images. Briefly, the camera was mounted on a tripod, and LED lighting was used to control for variations in ambient lighting, providing uniform illumination across all teeth. Two images were taken for each participant. The first image was captured before dye application. The erythrosine dye (in-house preparation, Faculty of Dentistry, Srinakharinwirot University, Bangkok, Thailand) was then applied for 30 s to highlight dental plaque accumulation. After dye application, the second image was captured.

### 2.3 Image processing and data augmentation

**Figure 2:**
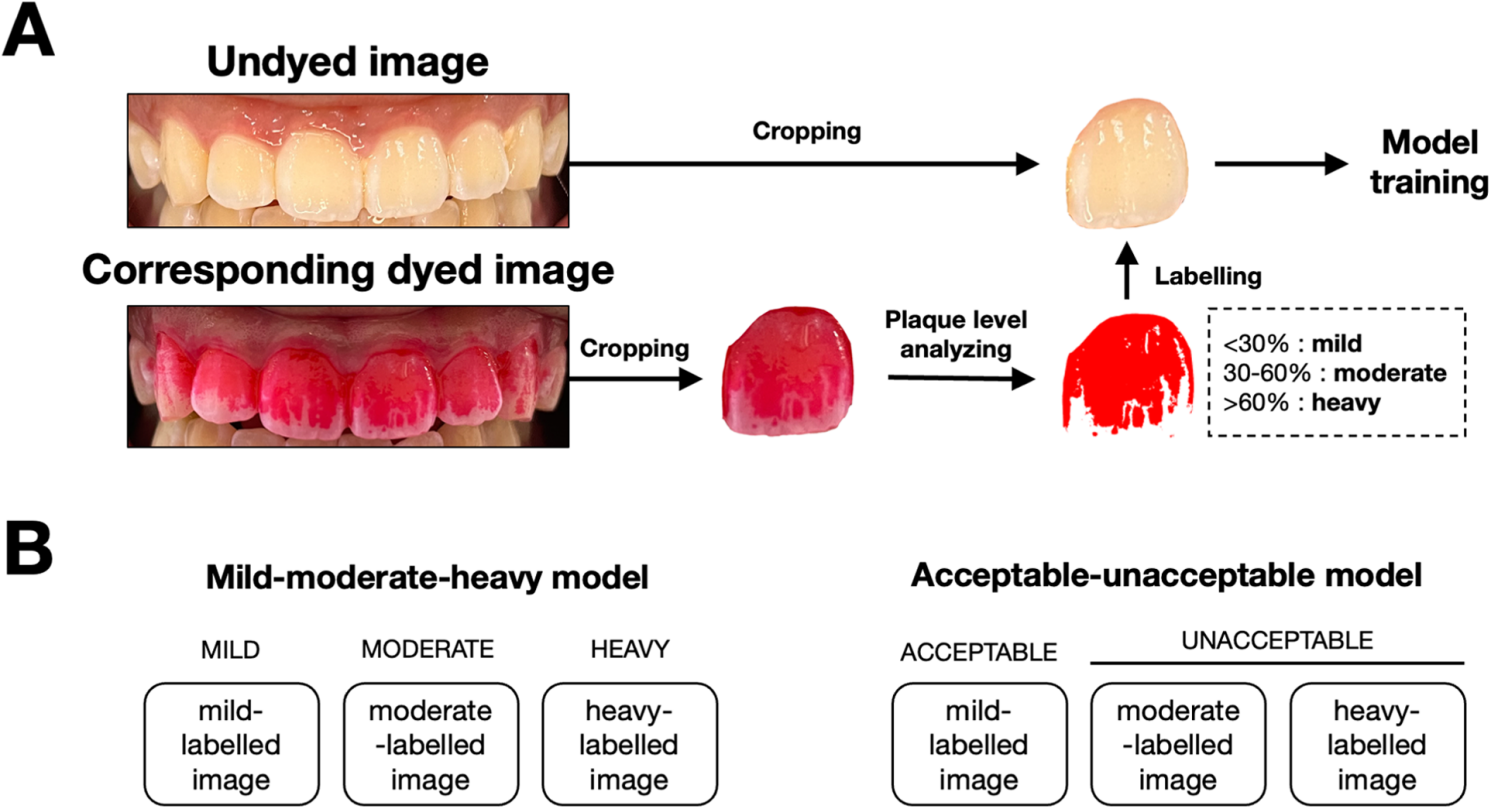
Image processing workflow and model classification framework for dental plaque detection. **(A)** Workflow for preparing images for model training. Undyed images were captured, followed by the application of a disclosing agent to reveal plaque. Dyed images were then cropped, and plaque coverage is analyzed. Based on the percentage of surface area covered by plaque, images were labeled into three categories: mild (<30%), moderate (30%–60%), and heavy (>60%). These labeled images were subsequently used to train the AutoML models. **(B)** Classification framework used for the two models. The mild-moderate-heavy model classifies images into three categories (mild, moderate, heavy), while the acceptable-unacceptable model simplifies the classification into two categories: acceptable (mild) and unacceptable (moderate and heavy).

In this study, we used single tooth images to train the model. Therefore, each image was cropped to isolate individual teeth using graphics editor application (Procreate version 5.3, Salvage Interactive, Hobart, Australia), resulting in six separate images for each participant. For each tooth, the images before and after plaque disclosure were paired. We used the dyed images as ground truth labels for model training. To achieve that, we analyzed the level of dental plaque on the dyed images depending on the percentage of the dyed area on the total labial surface of the tooth using ImageJ software (version 1.54 h, U.S. National Institutes of Health, Bethesda, MD). Briefly, the images were converted to an 8-bit grayscale format. The thresholding function was applied to isolate the dyed regions by setting values that distinguish plaque-stained areas from the rest of the image. The area measurement tool was then used to calculate the percentage of the dyed area. Tooth images with less than 30% dyed area were labelled as “mild”. Those with 30%–60% dyed area were labelled as “moderate”. Those with more than 60% dyed area were labelled as “heavy” (Figure 2). Data augmentation, including flipping and rotation, was used to balance and diversify the dataset.

### 2.4 Model development and training

The undyed tooth images were used for model training. The AutoML models were developed using the Vertex AI platform (M125 release with Gemini 1.5 Pro, Google Cloud, Mountain View, CA), according to the guidelines (9). Two datasets were created for single-label classification models. The first datasets included 3 groups (mild, moderate, heavy) as previously analyzed with ImageJ. The second datasets included 2 groups (moderate and heavy as “unacceptable”, mild as “acceptable”). The training pipeline was selected as “us-central1 (Iowa)”. The training options were set to high accuracy, 200–300 ms latency, and Google-managed encryption. The images in each dataset were randomly split for training (80%), validation (10%), and testing (10%) by the Vertex AI platform. The validation datasets were used to tune the model's parameters, monitor the performance, and prevent overfitting. After the model is optimized using the validation set, it is further evaluated on the test set, which was not used in training or validation.

### 2.5 Model evaluation

After models were developed, the Vertex AI AutoML models were evaluated. Statistical metrics were automatically analyzed by Vertex AI platform to assess the performance of the model, including area under precision-recall curve (AUPRC), precision value, recall value and, F1-score, as previously described (9).

## 3 Results

Two datasets were prepared for model training. The first dataset (for mild-moderate-heavy model) consisted of 100 mild-labelled images, 100 moderate-labelled images and 100 heavy-labelled images. The second dataset (for acceptable-unacceptable model) consisted of 196 acceptable-labelled images and 103 unacceptable-labelled images. Numbers of images in each group were set to meet the numbers suggested by Google Cloud and not lead to the excessive data augmentation. After the training and validation, mild-moderate-heavy model and acceptable-unacceptable model were created and evaluated.

### 3.1 Mild-moderate-heavy model

**Figure 3:**
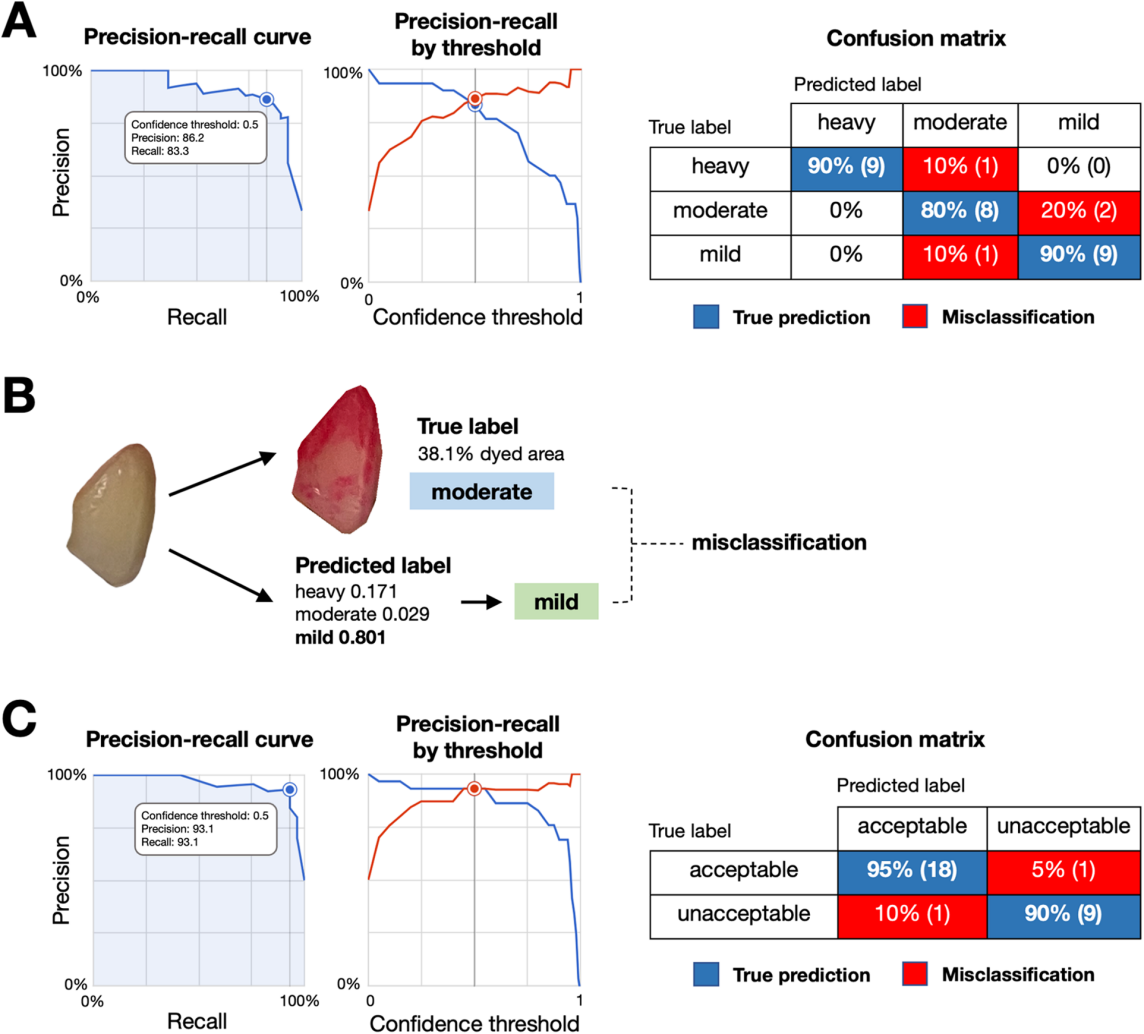
Performance and misclassification analysis of the plaque detection models. **(A)** Precision-recall curve (left) and precision-recall by threshold (middle) for the mild-moderate-heavy classification model, with a confusion matrix (right) showing true predictions (blue) and misclassifications (red) across categories. **(B)** Example of model misclassification. An image with a true moderate plaque label (38.1% dyed area) was misclassified as mild by the model, highlighting difficulties in distinguishing plaque levels with intermediate gradients. **(C)** Precision-recall curve (left) and precision-recall by threshold (middle) for the acceptable-unacceptable classification model, with a confusion matrix (right) showing true predictions (blue) and misclassifications (red).

The model was developed using 300 images, with 240 for training, 30 for validation, and 30 for testing. At a confidence threshold of 0.5, as shown in Table 1, the mild-moderate-heavy model achieved an average precision of 0.907, with the highest precision in the “heavy” category (0.983). The precision-recall curve and the confusion matrix were shown in Figure 3A.

**Table 1:** Performance metrics of the mild-moderate-heavy model and acceptable-unacceptable model.

Model	AUPRC	Precision	Recall	F1-score
Mild-moderate-heavy model	0.907	86.2%	83.3%	84.7%
Specific labels				
– Heavy	0.983	100%	90%	
– Moderate	0.886	80%	80%	
– Mild	0.836	80%	80%	
Acceptable-unacceptable model	0.964	93.1%	93.1%	93.1%
Specific labels				
– Acceptable	0.987	94.7%	94.7%	
– Unacceptable	0.956	90%	90%	

AUPRC, area under precision-recall curve (AUPRC).

### 3.2 Acceptable-unacceptable model

Although mild-moderate-heavy model showed the good precision value, we observed the relatively lower label-specific precision on moderate and mild categories. As images in moderate-labelled groups may have gradients or transitions which reportedly affecting the precision of the model (16). Given that mild levels of dental plaque deposits are preferable for the good plaque control. We performed second model training by categorizing the previously labelled images into 2 newly labelled groups, mild as “acceptable” and moderate and heavy as “unacceptable”. The second model was developed using 299 images, with 240 for training, 30 for validation, and 29 for testing. As shown in Table 1, the model achieved a precision of 0.964 and an F1 score of 93.1% at a 0.5 confidence threshold, demonstrating improved performance for practical clinical applications. The precision-recall curve and the confusion matrix were shown in Figure 3C.

## 4 Discussion

This study demonstrated the feasibility of using Vertex AI AutoML to detect dental plaque levels on permanent upper anterior teeth based on photographic images. AutoML models provided an accessible, automated approach to image classification without the need for extensive machine learning expertise. Recent studies have also explored machine learning approaches for dental plaque detection, with deep learning models applied to segmentation tasks. For example, Chen and colleagues developed deep learning models to detect dental plaque with high accuracy, though they required high-quality professional imaging (13). Similarly, You and colleagues utilized deep learning for detecting dental plaque on primary teeth using intraoral cameras (14). Unlike previous approaches, our study is among the first to employ AutoML platform using a smartphone camera taking standard photographic images from participants, to create classification model. This approach simplifies model development and makes it easier for both clinicians and patients to assess dental plaque status using accessible technology, such as smartphone cameras.

In the mild-moderate-heavy model, the overall performance was promising. However, the label-specific performance highlighted differences between categories (Table 1). These findings suggested that the model achieved high accuracy at detecting heavy dental plaque accumulations but struggled with moderate and mild cases. The misclassification was observed when the percentage of dyed area fell near the grouping thresholds, presenting challenges for accurate model development (Figure 3B). Misclassifications, particularly in the moderate and mild categories, are likely due to subtle changes, gradients, and transitional appearances in the images, making it difficult for the model to distinguish between classes. These findings associated with the moderate and mild categories are likely due to the nature of subtle changes, gradient and transition appearances in the images which make it difficult for the model to distinguish between classes. Similar challenges have been observed in other classification tasks as well (16).

To address these challenges, an acceptable-unacceptable model was developed. The second model demonstrated improved performance (Table 1), particularly for detecting unacceptable dental plaque levels, suggesting that binary classification is more suitable. The confusion matrix showed strong performance in detecting unacceptable levels of dental plaque, which is crucial for clinical decision-making in preventive care. The improved performance of the second model highlights the importance of choosing appropriate classification strategies based on the specific characteristics of the dataset. Given that near-zero dental plaque is preferred, simplifying the classification task can result in better overall model performance.

The large volume of data available for training helps AutoML models generalize better and achieve high accuracy across diverse cases (17, 18). In contrast, our study created a custom dataset by recruiting dental students and capturing high-quality photographs of their teeth. This approach enabled the dataset to reflect dental conditions more closely than those available in pre-existing image databases. However, while our custom dataset closely reflects real-world conditions, the limited number of images restricts generalization. Increasing the number of images will not only improve model generalization but also help the AutoML system handle more complex diversity. Future studies should focus on expanding the sample population to include greater variation, such as different ages, demographic profiles, dental conditions (e.g., orthodontic appliances, restorations), and tooth surfaces, as each presents unique dental plaque accumulation patterns. In addition, the inclusion of more diverse clinical settings would enable the AutoML model to better generalize across different lighting environments, various smartphone models, and variable image quality, ultimately increasing its applicability. While our current method of manually cropping individual teeth ensures precision, it may be inefficient in high-volume clinical settings. To address this, we are working on a new model that can assess dental plaque from multiple teeth in a single image, integrated with a user-friendly application. This advancement would make the technology more practical for clinical use, as well as enabling at-home monitoring and large-scale community screenings.

Despite these limitations, the study demonstrated the feasibility of using AutoML for real-world applications. Compared to manual detection techniques, which rely on the visual inspection of dental plaque or the use of disclosing dyes, the AutoML-based approach presents several advantages. It is non-invasive, eliminates the need for time-consuming manual assessments, and reduces reliance on dental plaque-disclosing agents. In addition, recent advancements allow AutoML model endpoints to be integrated into user-friendly applications, completing the process from image upload to evaluation within a few seconds (9). While the AutoML model-assisted process for evaluating dental plaque takes comparable amount of time to conventional dye-staining methods, it offers additional benefits, such as those previously mentioned. The simplicity of AutoML platforms allows researchers and clinicians without AI expertise to use these technologies. For example, they can integrate models into clinical tools or mobile apps and even develop their own models, bridging the gap between complex machine learning and everyday healthcare practices. As AI research in dentistry continues to grow, this study not only demonstrates the capability of AutoML to detect dental plaque levels but also highlights its potential to become part of future oral health management.

The results of this study underscore the clinical potential of AutoML in transforming dental plaque detection practices. By offering a digital method for assessing dental plaque levels, this technology could significantly enhance patient management. In clinical settings, faster and automated dental plaque assessments could improve the efficiency of dental professionals and reduce chair time. Moreover, the use of smartphone-compatible models makes this approach feasible for self-monitoring, empowering patients to take a more active role in their oral health. This capability could also be leveraged in large-scale community screenings, addressing oral health disparities, and facilitating preventive care in populations with limited access to traditional dental services.

## 5 Conclusion

In conclusion, the results of this study indicated that Vertex AI AutoML can be a valuable tool for image-based detection of dental plaque, with high accuracy in distinguishing between different levels of dental plaque. For dental professionals, this model offers an alternative workflow and reduces reliance on traditional dye-based methods. Additionally, its compatibility with smartphone opens new possibilities for remote monitoring and large-scale public health initiatives, paving the way for improved oral health outcomes on both an individual and community level. However, model performance can be affected by the complexity of the dataset. Future studies should focus on expanding datasets including more diverse patient populations and clinical settings and validating models in diverse clinical environments to enhance their generalizability and real-world applicability in preventive dental care.

## Data Availability

The original contributions presented in the study are included in the article/Supplementary Material, further inquiries can be directed to the corresponding author.
